# Progressions of the correlation between lipid metabolism and immune infiltration characteristics in gastric cancer and identification of BCHE as a potential biomarker

**DOI:** 10.3389/fimmu.2024.1327565

**Published:** 2024-01-31

**Authors:** Shibo Wang, Xiaojuan Huang, Shufen Zhao, Jing Lv, Yi Li, Shasha Wang, Jing Guo, Yan Wang, Rui Wang, Mengqi Zhang, Wensheng Qiu

**Affiliations:** ^1^ Department of Oncology, The Affiliated Hospital of Qingdao University, Qingdao, China; ^2^ Department of Dermatology, The Affiliated Hospital of Qingdao University, Qingdao, China

**Keywords:** gastric cancer, lipid metabolism, prognostic signature, TIICs, BChE, immunotherapy

## Abstract

**Background:**

Globally, gastric cancer (GC) is a category of prevalent malignant tumors. Its high occurrence and fatality rates represent a severe threat to public health. According to recent research, lipid metabolism (LM) reprogramming impacts immune cells’ ordinary function and is critical for the onset and development of cancer. Consequently, the article conducted a sophisticated bioinformatics analysis to explore the potential connection between LM and GC.

**Methods:**

We first undertook a differential analysis of the TCGA queue to recognize lipid metabolism-related genes (LRGs) that are differentially expressed. Subsequently, we utilized the LASSO and Cox regression analyses to create a predictive signature and validated it with the GSE15459 cohort. Furthermore, we examined somatic mutations, immune checkpoints, tumor immune dysfunction and exclusion (TIDE), and drug sensitivity analyses to forecast the signature’s immunotherapy responses.

**Results:**

Kaplan-Meier (K-M) curves exhibited considerably longer OS and PFS (p<0.001) of the low-risk (LR) group. PCA analysis and ROC curves evaluated the model’s predictive efficacy. Additionally, GSEA analysis demonstrated that a multitude of carcinogenic and matrix-related pathways were much in the high-risk (HR) group. We then developed a nomogram to enhance its clinical practicality, and we quantitatively analyzed tumor-infiltrating immune cells (TIICs) using the CIBERSORT and ssGSEA algorithms. The low-risk group has a lower likelihood of immune escape and more effective in chemotherapy and immunotherapy. Eventually, we selected BCHE as a potential biomarker for further research and validated its expression. Next, we conducted a series of cell experiments (including CCK-8 assay, Colony formation assay, wound healing assay and Transwell assays) to prove the impact of BCHE on gastric cancer biological behavior.

**Discussion:**

Our research illustrated the possible consequences of lipid metabolism in GC, and we identified BCHE as a potential therapeutic target for GC. The LRG-based signature could independently forecast the outcome of GC patients and guide personalized therapy.

## 1 Introduction

Global data in 2020 displays about 1.09 million new instances reported and 769,000 deaths of gastric cancer (GC), placing fifth in prevalence rate and fourth in fatality among malignant tumors (1). Although emerging treatments like targeted therapy and immunotherapy have demonstrated significant promise in improving the prognosis of advanced GC (2), their overall treatment efficacy is still lacking, and the 5-year survival rate presents a challenge (3).

Lipids, including phospholipids, fatty acids (FA), triglycerides, and cholesterol, are widely distributed in human tissues and organs (4). Lipid metabolism disorders can induce and infiltrate various chronic diseases, such as atherosclerosis, obesity, diabetes, and cancer (5–8). One of the most critical characteristics of cancer is lipid metabolic reprogramming, which is vital for encouraging the derivation and progression of cancer and remodeling TME. Among that, abnormal cholesterol metabolism is one of the most common pathways. Cholesterol can encourage tumor proliferation by activating the Hedgehog and mTORC1 signaling pathways (9), and promote tumor metastasis through epithelial-mesenchymal transition (EMT) (4). Reprogramming cholesterol metabolism in tumor cells or TIICs may influence tumor immune recognition and facilitate immune escape.

Moreover, FA metabolism reprogramming also is a significant process in cancer progression (10). The main enzymes in FA synthesis include sterol regulatory element binding protein (SREBP), fatty acid synthase (FASN), and acetyl CoA carboxylase (ACC). SREBP-1 and SREB-2 expression levels are markedly elevated in glioblastoma and prostate cancer, and ACC is upregulated in head and neck squamous cell carcinoma (11–13). Overexpression of FASN in breast cancer (BC) leads to chemoresistance, and improving chemotherapy-susceptibility can be achieved through targeted suppression of FASN (14–16). CPT1α, or carnitine palmitoyl transferase, is the primary enzyme involved in the catabolism of FA. Inhibiting the activity of CPT1α can decrease FA β- Oxidize, thereby reducing energy supply to tumor cells and exerting anti-tumor effects (17, 18). Furthermore, lipid droplets are formed in the endoplasmic reticulum when cellular lipids are excessive (19), which provide sufficient energy for cancer metastasis (20).

Over the past decade, immune checkpoint inhibitors (ICIs) have substantially improved clinical immunotherapy for GC (21, 22). There is mounting evidence that the tumor microenvironment (TME) significantly influences cancer growth and induces chemotherapy and immunotherapy resistance, and tumor-infiltrating immune cells (TIICs) have been demonstrated to assist in the advancement of cancer (23, 24). For instance, regulatory T cells (T-reg cells) have vital immunosuppressive functions, which regulate the activation and differentiation status of CD4+ or CD8+T cells and impede anti-tumor immune defenses, boosting tumor development and metastasis (25–27). Nevertheless, there is still a lack of comprehensive investigation into the relationship between gastric cancer, TME, and lipid metabolism.

This study utilized lipid metabolism-related genes (LRGs) to construct a predictive signature for GC, which has a reliable predictive effect on OS and PFS. Analyze the potential mechanism of this model through functional enrichment and further illuminate its relationship with immune infiltration characteristics. Additionally, we also forecast the immunotherapy effectiveness of GC persons. In summary, this investigation offers new directions for locating viable prognostic biomarkers and more efficient treatment options for GC.

## 2 Materials and methods

### 2.1 Data collection

The Cancer Genome Atlas (TCGA) database (https://portal.gdc.cancer.gov/) and the Gene Expression Omnibus (GEO) database (https://www.ncbi.nlm.nih.gov/) were the sources of the RNA-sequencing data and the associated clinicopathological data. The TCGA-STAD cohort represents 36 normal and 410 cancer specimens, and the GSE15459 cohort includes 192 cancer samples. A total of 13 gene sets, and 895 LRGs were derived from the Molecular Signatures Database (MisgDB) website (http://www.gsea-msigdb.org/gsea/msigdb/index.jsp). **Supplementary Table S1** lists specific gene sets and corresponding genes. The copy number variation (CNV), somatic mutation, and RNA stemness scores (RNAss) were attained from the UCSC Xena database (https://xenabrowser.net/datapages/). Download information on immunotherapy from The Cancer Immunome Atlas (TCIA, https://tcia.at/patients). This study excluded samples from TCGA-STAD with a survival period of ≤ 30 days. Subsequently, 366 GC specimens were categorized into training and testing sets (n = 183 for each) at random (28). The lipid metabolism-based prognostic signature was constructed using the training set, while the test set and GSE15459 set were applied as the validation set.

### 2.2 Differential expression analysis

Based on the predetermined cutoff parameters, the “limma” package determined the differentially expressed genes (DEGs) between tumor and standard specimens. Afterward, DELRGs were discovered by intersecting DEGs with LRGs. The functional and pathway enrichment analysis was also undergone on these DELRGs using the Gene Ontology (GO) and Kyoto Encyclopedia of Genes and Genomes (KEGG).

### 2.3 Development and verification of the lipid metabolism-related prognostic signature

We first employed univariate Cox analysis to determine OS-related LRGs and evaluate their CNV alterations. The optimal LRGs-based signature was created using LASSO and multivariate Cox regression analyses. The following explains how the risk scores were calculated:


$$\text{Risk score}=\sum_{\text{i}=1}^{\text{n}}\left(\text{Coefi}*\text{Expi}\right)$$

The terms “n,” “Coefi,” and “Expi” signify the number of signature genes, coefficients, and gene expression levels, respectively.

According to the median risk score, all specimens were separated into different risk subgroups. We plotted the K-M curves using the “survival” and “survmine” packages to demonstrate the effectiveness of the signature for forecasting OS. The reliability of the risk signature was appraised by the receiver operating characteristic (ROC) curves and validated via the testing and GSE15459 sets. Moreover, principal component analysis (PCA) was visualized for dimensionality reduction to analyze the signature’s capacity for grouping.

### 2.4 Functional enrichment analyses

GO and KEGG enrichment analyses were carried out utilizing the “clusterProfiler” package to derive biological processes and signal pathways (q-value< 0.05). Gene Set Enrichment Analysis (GSEA) (29) has also been employed to screen for potential mechanistic pathways between risk categories. The reference molecular database chosen was “c2.cp.Kegg.Hs.symbols.gmt” [KEGG], and |NES| > 1 and q-value< 0.1 were deemed statistical meaningful.

### 2.5 Construction and assessments of a nomogram

In both the TCGA and GSE15459 cohorts, univariate and multivariable Cox analyses were employed to determine if risk scores could function as a meaningful predictive element (p<0.05). Utilizing the R “rms” and “regplot” packages, we designed a clinical-related nomogram according to risk signature and other clinicopathological characteristics (30). Afterward, we generated calibration and ROC curves to verify the nomogram’s capacity for prediction.

### 2.6 Relationships of the signature with tumor environment and tumor-infiltrating immune cells

We employed the ESTIMATE algorithm (31) to determine stromal scores (the content of stromal cells), immune scores (the content of immunity cells), and tumor purity (the number of tumor cells) in the TME. Besides, the abundance of 22 different types of TIICs was measured by applying the LM22 signature and CIBERSORT algorithms (32). Single-sample gene set enrichment analysis (ssGSEA) scores were computed by the “GSVA” package (33) to gauge the level of TIICs in each tumor sample. We then employed the Wilcoxon test to explore the discrepancies in immune cell compositions between LR and HR classes. The association between risk scores and partial TIICs was performed by Spearman analysis.

### 2.7 Immunotherapy prediction and chemotherapy drug sensitivity

We downloaded somatic mutation data and calculated tumor mutation burden (TMB) values. The somatic mutation landscape was depicted via waterfall plots. We inspected the values of immunological checkpoints, ICIs, RNAss, and TIDE (http://tide.dfci.harvard.edu/) to anticipate gastric cancer individuals’ clinical immune therapy efficacy. The “oncoPredict” package was employed to examine the sensitivity of standard chemotherapy medications. The IC50 value demonstrates drug sensitivity.

### 2.8 Human protein atlas

Human normal and cancerous tissue protein expression data were available in the HPA database (https://www.proteinatlas.org/) (34). This study validated the signature genes’ protein levels between gastric cancer and normal tissues through immunohistochemistry (IHC) in the HPA database.

### 2.9 Quantitative real-time polymerase chain reaction

The human gastric epithelial cell line (GSE-1) and two human GC cell lines (HGC-27, AGS) used in this study were furnished by the Chinese Academy of Sciences. Using TRIzol® reagent extract RNA from various cells and reverse transcribed into cDNA. 1 µl of reverse transcription product, 3.6µl of DEPC, 5 µl of SYBR, and 0.2 µl each of forward and reverse primer were used in the qRT-PCR procedure. The amplification program for qRT-PCR was configured as follows: 95°C 3 minutes, followed by a cycle of 95°C 30 seconds, 55°C 20 seconds, and finally 72°C 20 seconds. Draw the PCR product’s melting curve (95°C for 15 seconds, 60°C for 15 seconds, and 95°C for 15 seconds) after the amplification reaction. All samples used GAPDH as the internal reference, and the 2^-ΔΔCT^ approach was applied to compare the expression levels of five signature genes. In GraphPad Prism 9, One-way ANOVA was utilized to detect statistical differences in five signature genes between gastric cancer and gastric epithelial cells. The primers used are as follows: APOA1-F, 5’-GACAGCGTGACCTCCACCTTC-3’ and APOA1-R, 5’-CTTCACCTCCTCCAGATCCTTGC-3’. BCHE-F, 5’-AAGCCATTCATTGTTCACCAGAGC-3’ and BCHE-R, 5’-AAAGAGATGTTACCGCCCAAGGAG’. CYP19A1-F, 5’-CACATCTGGACAGGTTGGAGGAG-3’ and CYP19A1-R, 5’-AGCATGACACGACGCAGAAGG-3’. PLA1A-F, 5’-GACGCTGTCTGGATTGCTTTAACC-3’ and PLA1A-R, 5’-CCACCTTGTTCCACCAGTCCTATC-3’. STARD5-F, 5’-GGAGGTGTGGGACTGTGTGAAG -3’ and STARD5-R, 5’-AGCGGAGGGAGTGGAGGTTC-3’. GAPDH-F, 5’-TGCACCACCAAC TGCTTAGC-3’and GAPDH-R, 5’-GGCA TGGACTGTGGTCATGAG-3’.

### 2.10 Construction of RNAi lentivirus vector

Construction of RNAi lentivirus vector by the Gikai gene (http://www.genechem.com.cn/). The name of the target lentiviral vector: GV493. Element sequence: hU6-MCS-CBh-gcGFP-IRES-puromycin. RNAi negative control (sh-NC) sequence: TTCTCCGAACGTGTCACGT. The shRNA sequence designed for BCHE is as follows: sh-BCHE-1: CCTTGAATACAGAGTCAACAA. sh-BCHE-2: CCAGACATATTACTTGAACTT. Transfect AGS and HGC-27 cell lines under the guidance of RNAi lentivirus vector construction and packaging manual (version 5.0).

### 2.11 Western blotting

Total proteins were extracted from corresponding cell lines using RIPA lysate (Beyotime, Shanghai, China), separated by sodium dodecyl SDS-PAGE, and transferred to the PVDF membrane. Subsequently, the membrane was incubated with 5% milk at room temperature for 1 hour and then incubated overnight with an appropriate primary antibody at four °C. Afterward, we used horseradish peroxidase-conjugated secondary antibody to visualize the target proteins. The membranes were washed with TBST both before and following antibody incubation. This article uses the antibodies listed below: BCHE (Proteintech, Wuhan, China) and β- Actin was purchased from Abcam. β- Action as the internal reference.

### 2.12 CCK-8 assay

Inoculate GC cells transfected with sh-NC or sh-BCHE lentivirus into a 96-well plate. Measure the OD value at a wavelength of 450 nm using the Cell Counting Kit-8 (Beyotime, Shanghai, China) according to the manufacturer’s operating guidelines. Monitor cell proliferation every 24 hours for a period of 3 days used to evaluate cell proliferation.

### 2.13 Colony formation assay

Place AGS and HGC-27 cell lines transfected with sh-NC or sh-BCHE in 6-well plates with 1000 cell densities. Subsequently, these cells cultured in RMPI-1640 medium containing 10% fetal bovine serum for 10 days. We used methanol for fusion and colony proliferation, and stained and proliferated the colonies with 1% crystal violet (Beyotime, Shanghai, China), and finally took photos of the colonies.

### 2.14 Wound healing and transwell assays

We inoculated GC cells transfected with sh-NC or sh-BCHE lentivirus into a 6-well plate and cultured them to a sub-fusion state (90-100%). Create a linear scratch wound using a 200-µL pipette tip and obtain images at 0 and 24 hours. Measure the scratch area three times and evaluate the cell healing rate. For the transwell assay, we inoculated cells at a density of 2×105 into the upper chamber (Corning, New York, USA) and cultured with 200μl of serum-free medium. Add 600μl medium containing 10% fetal bovine serum to the lower chamber and incubate at 5% CO2 37 °C for 24 hours. Then, the cells that migrated to the bottom of the filter were fixed with 4% paraformaldehyde for 20 minutes, stained with 0.5% crystal violet dyes, and photographed under an inverted phase contrast microscope (Motic).

### 2.15 Statistical analysis

We employed R software (Version 4.2.2, http://www.R-project.org) and GraphPad Prism software (Version 9.3.1, CA, USA) to conduct all statistical analyses. Partial data was processed by Perl software (Version 5.30.0.1, https://strawberryperl.com/). K-M curves were evaluated for various risk groupings’ prognoses. The ROC and DCA curves were performed to assess the pragmatic capabilities of the risk signature and nomogram model. Cox regression analyses were exploited to pursue meaningful risk factors for GC. Wilcoxon rank-sum test and Spearman analysis were applied for comparative and correlation analyses between risk subgroups. P< 0.05 was regarded as the threshold for statistical significance (ns: p > 0.05, *: p ≤ 0.05, **: p ≤ 0.01, ***: p ≤ 0.001).

## 3 Results

### 3.1 Identification of differentially expressed lipid metabolism-related genes

**Figure 1:**
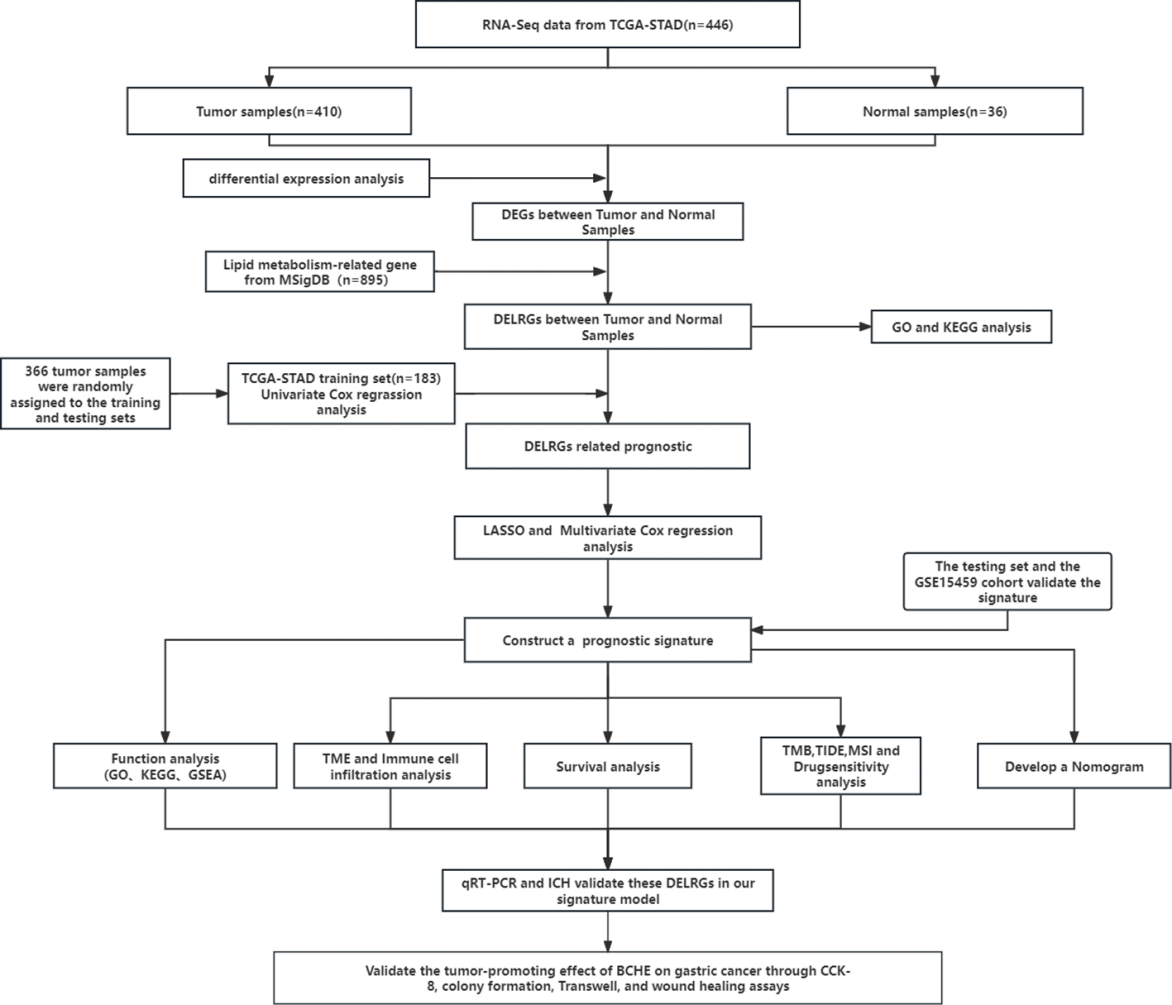
The flowchart of this research.

**Figure 2:**
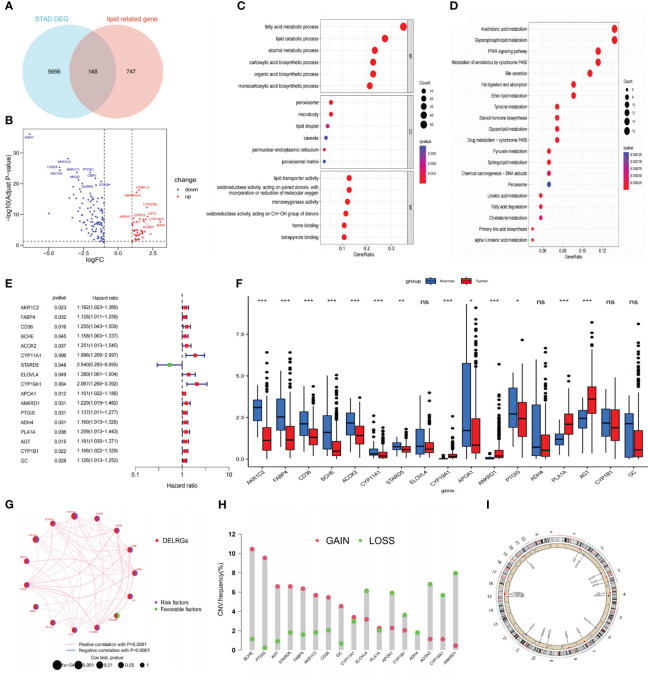
Identifying DELRGs with prognostic value. **(A)** Veen diagram between DEGs and LRGs in the TCGA cohort. **(B)** Volcano maps of 148 DELRGs. **(C, D)** GO and KEGG analyses of these DELRGs. **(E)** Univariate Cox regression analysis identified 17 OS-related DELRGs. **(F)** Expression of OS-related DELRGs in tumor and normal samples of the TCGA cohort. **(G)** The interaction network of these OS-related DELRG. **(H)** CNVs alteration in OS-related DELRGs in GC specimens. **(I)** Chromosomal locations of CNV alterations in OS-related DELRGs. *P< 0.05; **P< 0.01; ***P< 0.001. DEGs, differentially expressed genes; TCGA, The Cancer Genome Atlas; DELRGs, differentially expressed lipid metabolism-related genes; GO, Gene Ontology; KEGG, Kyoto Encyclopedia of Genes and Genomes; OS, overall survival; CNV, copy number variation; GC, gastric cancer. ns: p>0.05.

**Figure 1** depicts this study’s flowchart. Based on the |log FC| > 1 and p<0.05 criteria, 5814 DEGs were discovered in the TCGA-STAD database. Subsequently, we discovered 148 DELRGs (39 up-regulated and 109 down-regulated) by DEGs intersected with lipid metabolism-related genes (LRGs) (**Figures 2A, B**). Afterward, we further carried out GO and KEGG analyses to investigate these DELRG’s possible mechanisms. Not surprisingly, these genes were primarily implicated in fatty acid metabolic, lipid catabolic, steroid metabolic, and lipid transporter activity (**Figure 2C**). Furthermore, KEGG analysis displayed that these DELRGs were predominantly associated with the “PPAR signaling pathway,” “glycerophospholipid,” and “arachidonic acid “ metabolisms (**Figure 2D**), all of which were significantly associated with inflammation and cancer emergence.

### 3.2 Establish a lipid metabolism-related prognostic signature

In the TCGA-STAD cohort, researchers collected 366 cancer samples, excluding samples with a survival period of under 30 days. They were randomly separated into training and testing sets (n = 183 for each) for further study. The LRGs-based prognostic signature model was built using the training set (**Supplementary Table S2**). Initially, we screened out 17 DELRGs related to OS using univariate Cox regression analysis to establish each DELRG’s potential predictive value (**Figure 2E**). **Figure 2F** displayed OS-related gene expression levels in tumor and normal tissues. CYP1B1, ADH4, ELOVL4, and GC did not differ statistically significantly. Most of these genes were risk factors for gastric cancer, and the regulatory network showed that they were generally positively associated (**Figure 2G**).

**Figure 3:**
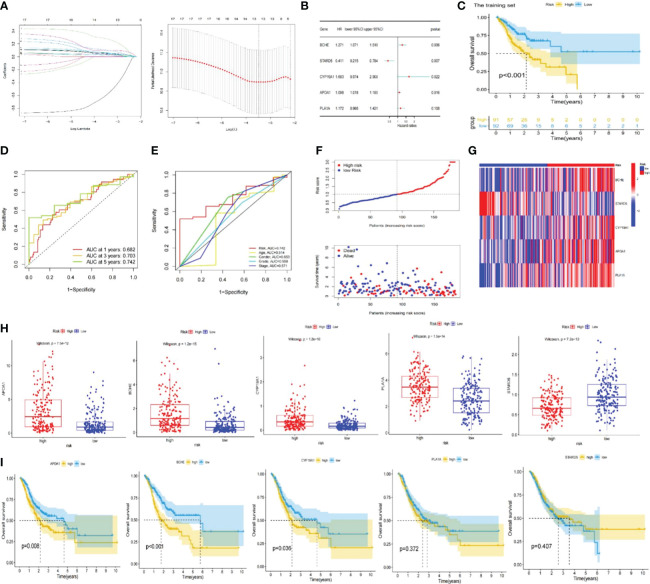
Construction of the lipid metabolism-based risk signature. **(A)** Lasso Cox regression analysis and cross-validation. **(B)** Multivariate Cox regression analysis for identifying the optimum signature genes. **(C)** Kaplan-Meier curve of high and low-risk subgroups in the training set. **(D)** ROC curves of the risk signature for predicting 1, 3, and 5-year survival. **(E)** ROC curve for predicting 5-year survival based on risk signatures and other clinical characteristics. **(F, G)** Distributions of risk scores, survival statuses and gene expression. **(H)** Expression of signature genes in high and low-risk subgroups in the entire TCGA set. **(I)** Kaplan-Meier curves of signature genes in the entire TCGA set. ROC; receiver operating characteristic; TCGA, The Cancer Genome Atlas.

Moreover, these 17 genes exhibited a high frequency of CNVs, with GAIN occurring more frequently than LOSS. ANKRD1 had the most significant loss, and BCHE had the highest gain (**Figure 2H**). The chromosomal locations of these CNV-altered genes are shown in **Figure 2I**. According to **Figure 3A**, we performed Lasso Cox analysis and cross-validation to avoid the signature from overfitting. Ultimately, we conducted a multivariate Cox regression analysis to ensure the stability and optimality of the signature. Five pivotal lipid metabolism-related genes—APOA1, BCHE, CYP19A1, PLA1A, and STARD5—were discerned and utilized to create the prognostic signature (**Figure 3B**). Consequently, each sample’s risk score was determined by its Cox coefficient and gene expression as: Risk score= (0.24012*BCHE expression) +(-0.88977*STARD5 expression) +(0.52063*CYP19A1 expression) +(0.09367*APOA1 expression) +(0.15832*PLA1A expression).

The training set’s median risk score was employed to categorize all GC samples into high- or low-risk groupings. **Supplementary Table S3** contains the risk score results.

### 3.3 Assessment and verification of the risk signature

**Figure 4:**
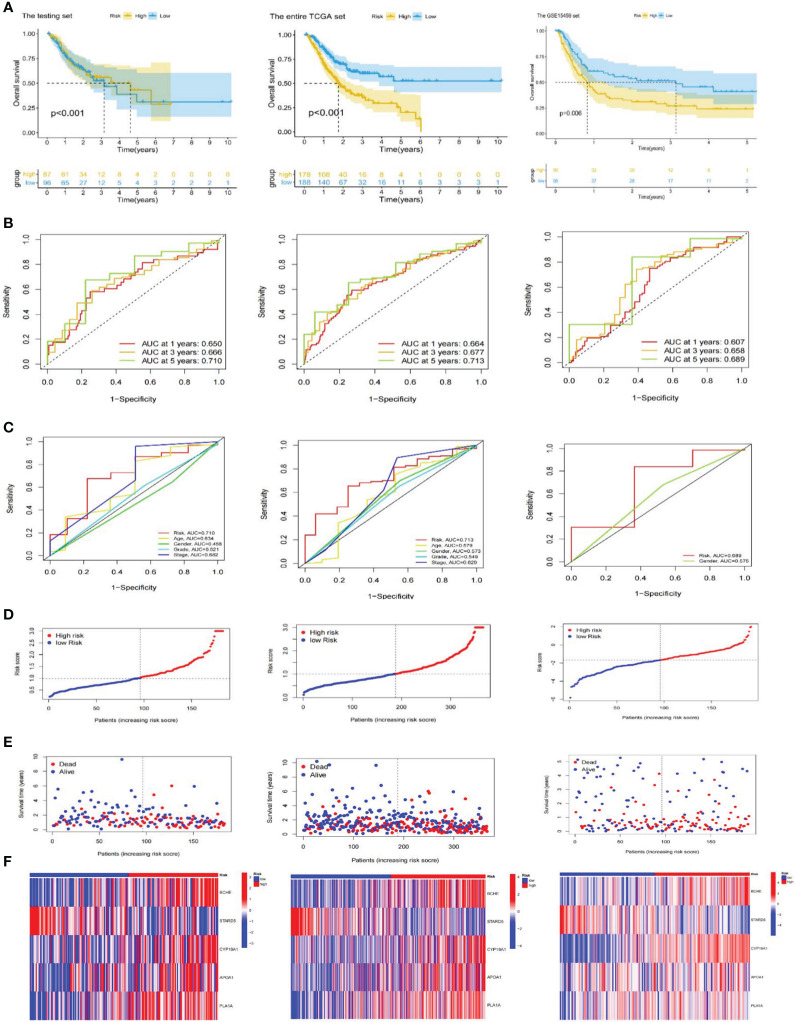
Validation of the risk signature. **(A)** Kaplan-Meier curves for high and low-risk subgroups in the testing set, the entire TCGA and GSE15459 cohorts. **(B)** ROC curves of the risk signature for predicting 1, 3, and 5-year survival in the testing set, the entire TCGA and GSE15459 cohorts. **(C)** ROC curve for predicting 5-year survival based on risk signatures and other clinical characteristics in the testing set, the entire TCGA and GSE15459 cohorts. **(D–F)** Distributions of risk scores, survival statuses and gene expression in the testing set, the entire TCGA and GSE15459 cohorts. TCGA, The Cancer Genome Atlas; ROC; receiver operating characteristic.

In the TCGA-STAD and GSE 15459 cohorts, K-M analysis displayed that the OS of the LR subgroup was considerably longer than the HR subgroup (**Figures 3C**, **4A**). **Figures 3D**, **4B** exhibited the AUC values of risk signature 1, 3, and 5-year survival ROC curves. By contrasting the 5-year ROC curve with additional clinical characteristics, the superiority of the signature was even more evident (**Figures 3E**, **4C**). We also plotted the distribution of risk scores, survival status, and time in the training group. The findings indicated that persons in the HR subgroup had a poor prognosis and validated in other sets (**Figures 3F**, **4D, E**). In addition, the heatmaps manifested the expression differences of signature genes in LR and HR groups (**Figures 3G**, **4F**). Finally, we generated boxplots and K-M curves of each signature gene to further excavate their expression levels and independent prognostic ability. As shown in **Figures 3H**, APOA1, BCHE, CYP19A1, and PLA1A were prominently expressed in the high-risk subgroup, while STARD5 was significantly lower. In K-M analysis, the low expression groups of APOA1, BCHE, and STARD5 exhibited longer OS, but the expression levels of PLA1A and STARD5 did not significantly impact the OS of GC patients (**Figure 3I**).

**Figure 5:**
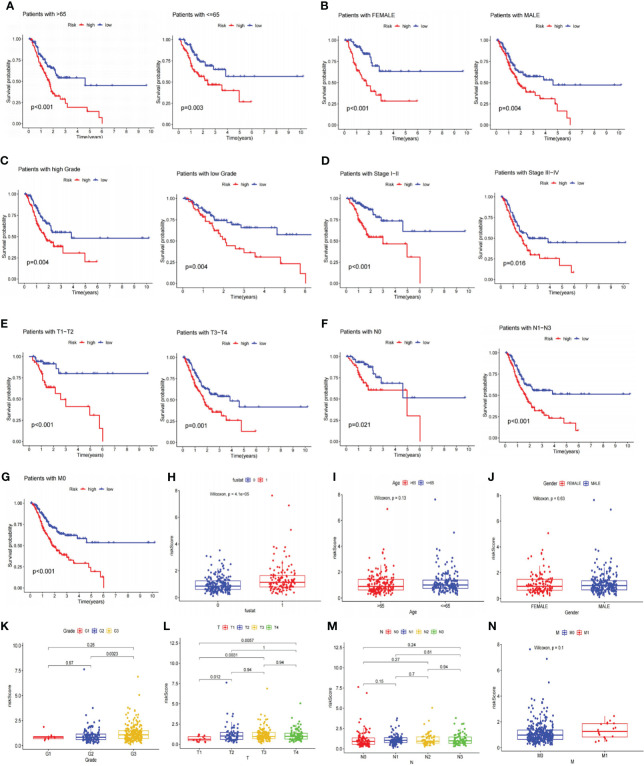
Analyses of the relationship between the risk signature and clinicopathological parameters. **(A–G)** The overall survival between the high and low-risk subgroups from the TCGA database are shown classifying patients by age, gender, tumor grade, clinical stage, T stage, N stage and M0 stage. Low-risk patients display longer overall survival than high-risk patients. **(H–N)** Systematic evaluation of risk scores and clinical parameters for GC samples, including survival status, age, gender, tumor grade, T stage, N stage, and M stage. TCGA, The Cancer Genome Atlas; GC, gastric cancer.

After that, we explored the LRGs-based signature’s capacity for prediction stratified based on different clinicopathological parameters in the TCGA database. The K-M curves suggested that patients in LR group had considerably better outcome than that of HR group in all categories fragmented by Age (>65/<=65; **Figure 5A**), Gender (female and male; **Figure 5B**), Grade (low/high; **Figure 5C**), Stage (I-II/III-IV; **Figure 5D**), T stage (T1-T2/T3-T4; **Figure 5E**), N stage (N0/N1-N3, **Figure 5F**), and M stage (M0, **Figure 5G**). Furthermore, we investigated the association between risk scores, clinicopathological features, and patient outcomes. The results exhibited that greater pathological Grade, T stage, and death (fustat=1) populations had considerably higher risk scores (**Figures 5H, K, L**). Nevertheless, there were no statistical differences in risk scores among Age, Gender, T-stage, and M-stage categories (**Figures 5I, J, M, N**).

### 3.4 Independent predictive significance of the risk signature

**Figure 6:**
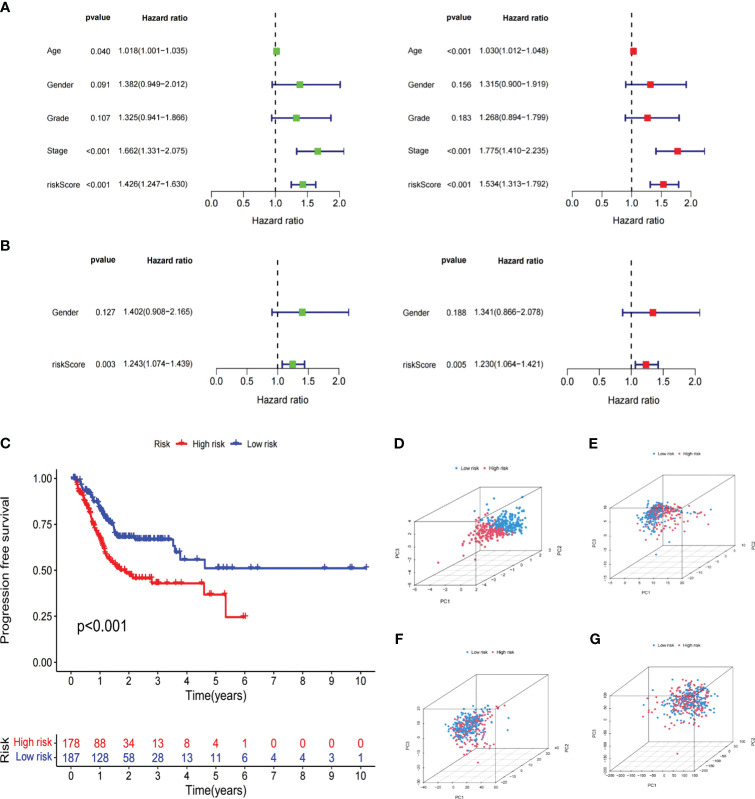
Independent verification of the risk signature. **(A)** Univariate and multivariate Cox regression analyses of the risk signature and clinical parameters in the TCGA cohort. **(B)** Univariate and multivariate Cox regression analyses of the risk signature and clinical parameters in the GSE15459 cohort. **(C)** The Kaplan-Meier curve shows the PFS of high and low-risk subgroups in the TCGA cohort. (**D–G**) PCA between low- and high-risk subgroups based on the lipid metabolism related signature, differently expressed lipid metabolic genes, all lipid metabolism-related genes, and the entire gene expression profiles. The Cancer Genome Atlas; PFS, progression free survival; PCA, principal components analysis.

To better demonstrate risk scores could independently predict prognosis, we first performed univariate Cox analysis in the TCGA cohort, finding that Age (HR=1.018,95% CI = 1.001-1.035, p<0.05), Stage (HR=1.662,95% CI =1.331-2.075,p<0.001) and Risk Score (HR=1.426,95% CI =1.247-1.630,p<0.001) were related to OS. Multivariate regression confirmed the independence of Age (HR=1.030,95% CI =1.012−1.048,p<0.001), Stage (HR=1.775,95% CI =1.410−2.235,p<0.001), and Risk Score (HR=1.534,95% CI =1.313−1.792,p<0.001) (**Figure 6A**). The GSE15459 set’s risk score P-value was less than 0.01 in both univariate and multivariate Cox analyses, further supporting the viability of using risk scores to predict prognosis independently (**Figure 6B**). Additionally, there was a statistically significant difference (p<0.001) in PFS, with persons in the LR subgroup having noticeably longer PFS (**Figure 6C**). PCA analysis revealed distinct dimensions among different subgroups based on signature genes, DELRGs, all LRGs, and the whole gene expression profiles (**Figures 6D-G**). Among that, the substantially diverse distribution directions for HR and LR groups were observed based on the risk signature (**Figure 6D**), confirming the efficacy of our prognostic signature in identifying various GC patients.

### 3.5 Functional enrichment analysis

**Figure 7:**
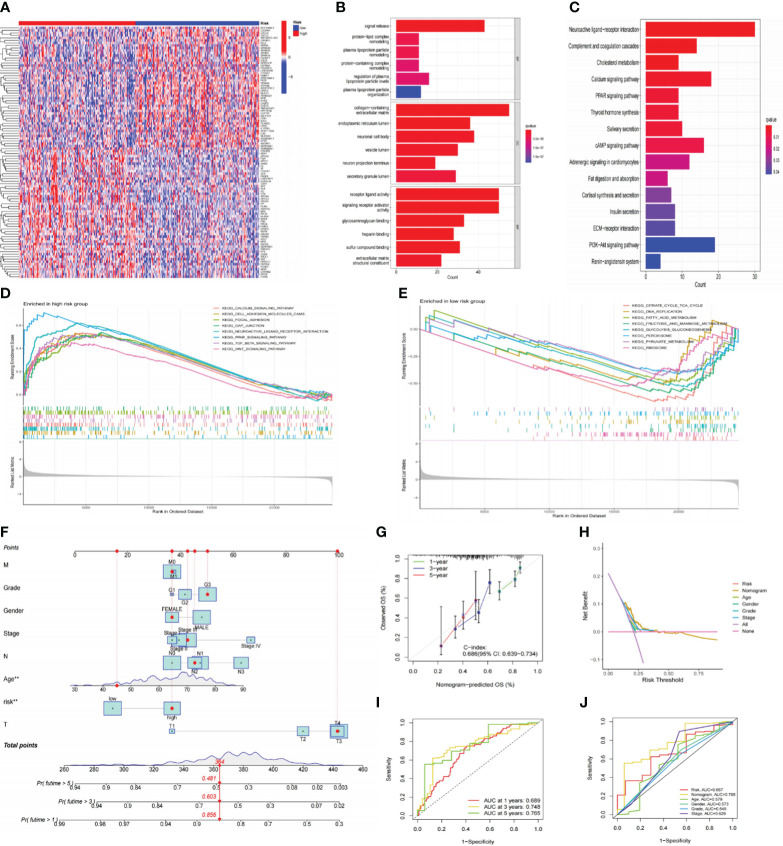
Functional analysis of risk signatures and construction of nomogram. **(A)** Heatmap of the top 100 DEGs between high and low-risk subgroups. **(B, C)** GO and KEGG analyses of DEGs between high and low-risk groups. **(D, E)** GSEA analysis of the primary enriched pathways in high and low-risk groups. **(F)** The nomogram constructed based on the risk signature and other clinicopathological parameters. **(G)** Calibration plots for nomograms at 1, 3, and 5-years. **(H)** DCA of the nomogram, risk signature, and other clinicopathological parameters. **(I)** ROC curve of the risk signature for predicting 1, 3, and 5-year survival. **(J)** ROC curve for predicting 5-year survival based on the nomogram and other clinical characteristics. DEGs, differentially expressed genes; GO, Gene Ontology; KEGG, Kyoto Encyclopedia of Genes and Genomes; GSEA, gene set enrichment analysis; DCA; decision curve analysis; ROC, receiver operating characteristic.

Originally, we performed differential gene analysis (|log FC| > 1 and p< 0.05) across two risk groupings, yielding 544 DEGs (496 up-regulated and 48 down-regulated in the high-risk subgroup). In **Figure 7A**, we plotted a heatmap of the most prominent 100 DEGs. On these DEGs, we then carried out enrichment analyses. GO analysis demonstrated that these DEGs were predominantly abundant in the “signal release” and “hormone transport” of biological processes (BP) and the collagen−containing extracellular matrix in cellular components (CC). Regarding molecular function (MF), DEGs exhibited an enrichment in receptor-ligand activity and signaling receptor activator (**Figure 7B**). Following KEGG analysis, these DEGs were preponderantly concentrated in the PI3K−Akt, Calcium, cAMP, and PPAR signaling pathways (**Figure 7C**), all associated with tumor development and metastasis. Additionally, GSEA analysis was utilized to anatomize functional distinctions between risk subgroups (**Supplementary Table S4**). The findings indicated that the LR subgroup had enriched metabolic pathways, including fatty acid metabolism, citrate cycle TCA cycle, and glycolysis gluconeogenesis (**Figure 7D**). On the contrary, the paths remarkably situated in the HR subgroup contained PPAR, TGF-β, Calcium, and WNT signaling pathways and matrix-activated related pathways like gap junction and focal adhesion (**Figure 7E**). Accordingly, we speculated that the worse outcome of individuals in the HR subgroup was related to activating these carcinogenic pathways and matrix pathways leading to tumor metastasis.

### 3.6 Development and evaluation of a nomogram

To improve the clinical practicality of our signature even more, we constructed a nomogram using risk scores and other clinicopathological characteristics to predict OS (**Figure 7F**). The accurate predictability of the nomogram was demonstrated by the calibration curve (C-index:0.686), consistent with its OS estimates, confirming the satisfactory predictive ability of the nomogram (**Figure 7G**). In order, the 1-, 3-, and 5-year ROC curves had area under the curve (AUC) values of 0.689, 0.748, and 0.765 (**Figure 7I**). Additionally, the DCA curve (**Figure 7H**) and ROC curve (**Figure 7J**) demonstrated that the nomogram outperformed the risk signature and other conventional clinicopathological parameters in forecasting the prognosis of GC. In conclusion, the effectiveness and reliability of our nomogram were elucidated from various aspects.

### 3.7 The relevance between immune infiltration and risk signature

**Figure 8:**
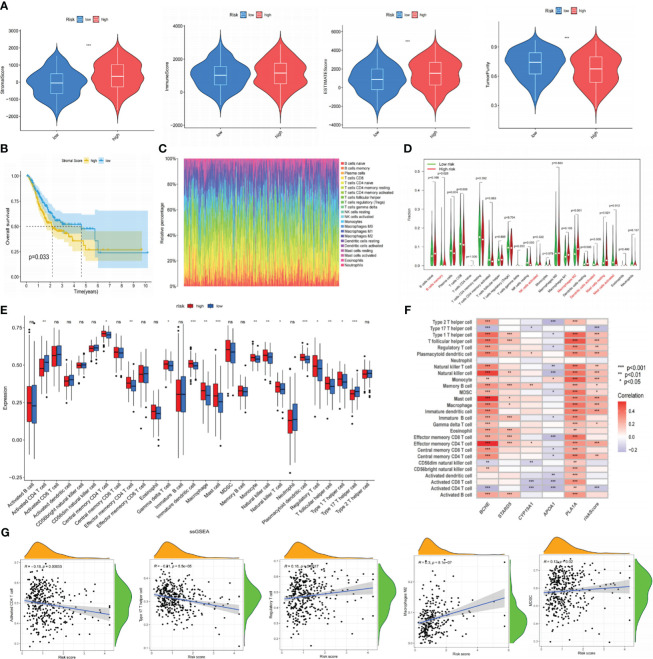
The correlation between immune infiltration and our risk signature. **(A)** The content of stromal cells, immune cells, and tumor purity in the TME of the TCGA cohort. **(B)** Kaplan-Meier curve indicates that patients with low stromal scores have longer OS. **(C)** The proportion of 22 types of TIICs in the GC population using the CIBERSORT algorithm. **(D)** Expression levels of 22 TIICs in high and low-risk groups. **(E)** Analyzing the infiltration levels of TIICs in high and low-risk groups using the ssGSEA algorithm. **(F)** Spearman correlation analysis between risk signature and TIICs. **(G)** Correlation analysis between risk scores and TIICs. *P< 0.05; **P< 0.01; ***P< 0.001. TME, tumor microenvironment; TCGA, The Cancer Genome Atlas; OS, overall survival; TIICs, tumor-infiltrating immune cells; GC, gastric cancer; ssGSEA, single-sample gene set enrichment analysis. ns: p>0.05.

The tumor microenvironment (TME) was known for its hypoxia, chronic inflammation, and immune suppression, and it was a crucial player in tumor development. We thus employed the ESTIMATE method to ascertain the fraction of tumor, stromal, and immune cells in the TME (**Supplementary Table S5**). According to the findings, the LR group had greater tumor purity scores, whereas the HR group had higher stromal and estimation values. Immune score differences were not statistically significant (**Figure 8A**). The K-M curve revealed that individuals with low stromal scores exhibited prolonged OS (**Figure 8B**). Next, we employed the CIBERSORT algorithm to measure the percentage of 22 TIICs in each tumor sample of the TCGA database. As shown in **Figures 8C, D**, the LR subgroup observed elevated infiltration of B memory cells, activated NK cells, and activated Dendritic cells, all of which exerted anti-tumor effects. Conversely, the high-risk group had more tumor-associated macrophages (TAMs; M2 phenotype) infiltration, which could release immunosuppressive cytokines and stimulate cancer invasion. Additionally, we utilized ssGSEA to investigate the connection between the risk signature and TIICs, discovering that most immunosuppressive cells were closely associated with the HR group (**Figures 8E, F**). The Spearman analysis showed that risk scores had a positive correlation with regulatory T cells, MDSC, and TAMs but a negative correlation with CD4+ and type 17 helper T cells (**Figure 8G**). These discoveries suggested that persons in the high-risk category might promote immune escape by inducing immune suppression, resulting in a worse prognosis. The results of ESTIMATE, CIBERSORT, and ssGSEA are all summarized in **Supplementary Table S5**.

### 3.8 Mutation analysis and immune efficacy prediction of the prognostic signature

**Figure 9:**
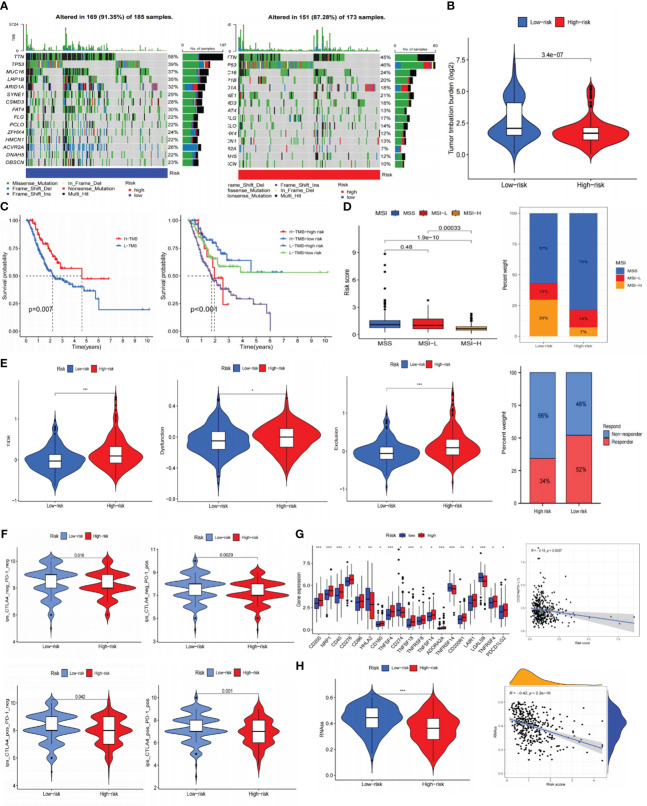
Prediction of immunotherapy for gastric cancer. **(A)** Somatic cell mutation frequencies in high and low-risk groups. **(B)** TMB levels in high and low-risk groups. **(C)** Kaplan-Meier curve of OS in high and low-TMB groups; Kaplan-Meier curve survival curves show different survival among the four groups that combined TMB with risk signature. **(D)** Analysis of risk signature and microsatellite state. **(E)** TIDE, dysfunction, exclusion, and immunotherapy response in high and low-risk groups. **(F)** Joint analysis of anti-PD-1 antibody and anti-CTLA-4 antibody in risk signature. **(G)** Expression of ICIs in high and low-risk groups, and the correlation between risk scores and PD-L1. **(H)** RNAss value in high and low-risk groups, and the association between risk scores and RNAss. *P< 0.05; **P< 0.01; ***P< 0.001. TMB, tumor mutational burden; OS, overall survival; TIDE, tumor immune dysfunction and exclusion; ICIs, immune checkpoint inhibitors; RNAss, RNA stemness scores.

Previous research has demonstrated a close correlation between somatic mutations and cancer development (35). These mutations were also crucial in formulating targeted treatments for cancer. As a result, we conducted somatic mutation analysis on the TCGA database. It was demonstrated that compared to the HR subgroup, the LR-scoring population had a greater mutation incidence (91.35% vs. 87.28%), and the most frequent mutation type was missense mutation, trailed by nonsense and frameshift deletion. The waterfall plot exhibited that TTN, TP53, MUC16, LRP1B, and ARID1A were the five most prominent genes with mutation frequency (**Figure 9A**). Subsequently, we calculated the TMB values for each specimen. The LR group appeared to have higher TMB scores (**Figure 9B**), and individuals with elevated TMB values maintained prolonged OS. Patients with low-risk and high-TMB scores had the best prognosis among the four groups, according to our analysis of the combination of TMB and risk scores (**Figure 9C**).

Afterward, we predicted the immune therapy response of GC patients by analyzing several common biomarkers. **Figure 9D** suggested significant differences across various microsatellite groupings, where the MSI-H category had considerably lower risk scores. The overlay graph demonstrated that the microsatellite instability (MSI) percentage was more significant in the LR group (43%). Moreover, we performed TIDE analyses and discovered that the HR group displayed a more significant proportion of immunological non-responders along with higher TIDE, exclusion, and dysfunction scores (**Figure 9E**). This implied that the HR group may be more susceptible to immunological escape and not responsive to immunotherapy.

Using immunotherapy data from TCIA, we additionally explored the connection between the risk signature and ICIs. Programmed cell death protein 1 (PD-1) positive, cytotoxic T lymphocyte-associated antigen-4 (CTLA-4) positive, and PD-1/CTLA-4 positive groups showed greater immunotherapy efficacy in the LR subgroup (**Figure 9F**). According to immune checkpoints analysis, the low-risk population displayed increased programmed cell death-ligand 1 (PD-L1) expression (**Figure 9G**), indicating a greater chance of benefiting from immunotherapy. Furthermore, it was intriguing to note that there is a negative association between risk scores and RNAss (**Figure 9H**) that the lower the risk score, the more distinct the stem cell characteristics of GC cells and the lower the level of cell differentiation.

### 3.9 Drug sensitivity analysis and signature genes validation

**Figure 10:**
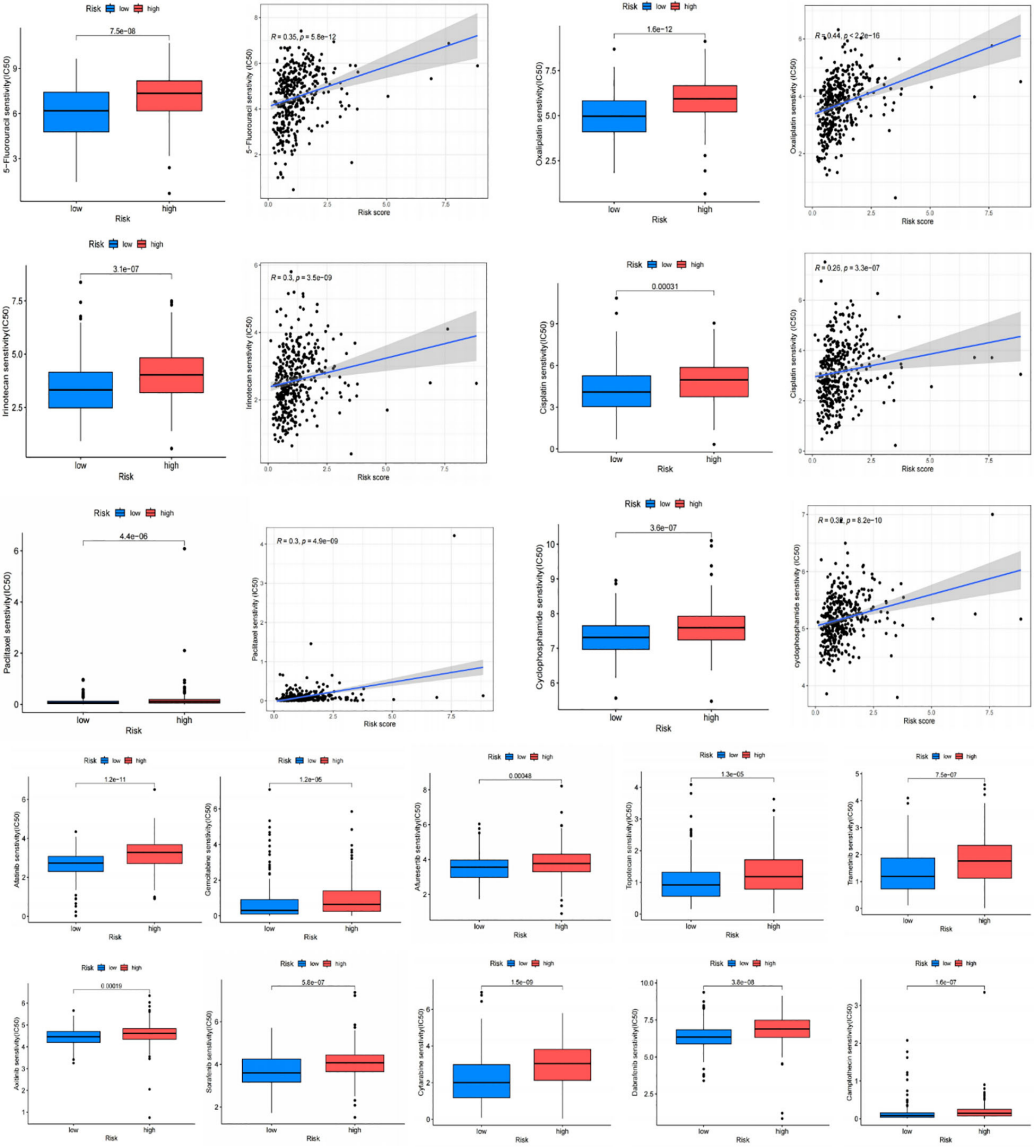
Drug sensitivity analysis. Chemotherapy drugs used for standard treatment of gastric cancer, such as oxaliplatin, cisplatin, paclitaxel, 5-fluorouracil, and irinotecan, are more effective in the low-risk group with lower IC50 values.

**Figure 11:**
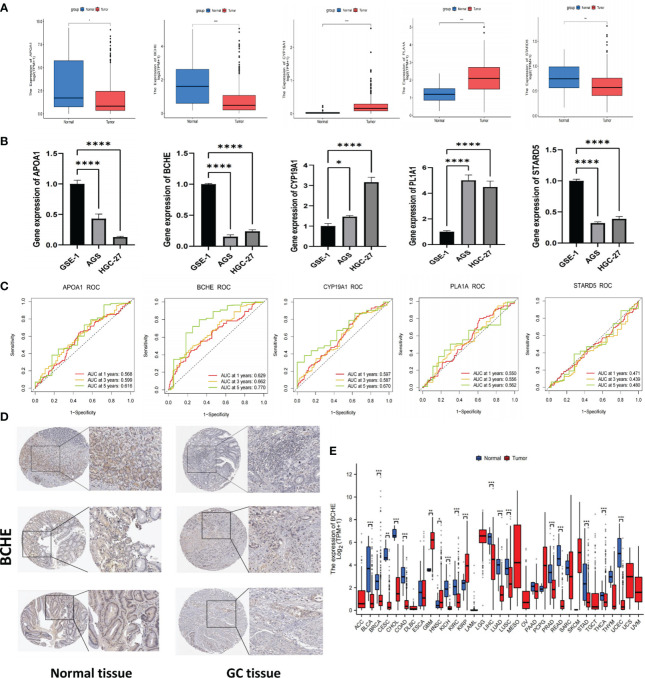
Verification of signature genes and Identifying BCHE as a potential biomarker. **(A)** Expression of APOA1, BCHE, CYP19A1, PL1A1, and STARD5 in tumor and normal samples of the TCGA cohort. **(B)** The qRT-PCR validation of signature genes. A human gastric epithelial cell line (GSE-1) and two human gastric cancer cell lines (AGS and HGC-27). **(C)** ROC curves of the five signature genes. **(D)** The protein expression of BCHE by HPA database. **(E)** Pan-cancer analysis of BCHE in TCGA database. *P< 0.05; **P< 0.01; ***P< 0.001. ****P< 0.0001. qRT-PCR, quantitative real-time polymerase chain reaction.

The IC50 of many common targeted drugs and standard chemotherapy drugs (such as 5-fluorouracil, oxaliplatin, cisplatin, and irinotecan) was positively correlated with risk scores based on the drug sensitivity analysis shown in **Figure 10**. This suggests that these drugs may benefit patients with low-risk scores more. Bioinformatics analysis revealed that APOA1, BCHE, and STARD5 were significantly expressed in normal samples, while CYP19A1 and PLA1A were considerably expressed in tumor samples in the TCGA-STAD cohort (**Figure 11A**). Subsequently, we employed qRT-PCR to confirm our signature’s dependability further (**Supplementary Table S6**). Consistent with the preceding analytical results, the expression of APOA1, BCHE, and STARD5 in human gastric cancer cells (AGC, HGC-27) was significantly lower (p<0.0001) compared to normal gastric epithelial cells (GSE-1). In contrast, the expression of CYP19A1 and PL1A1 was significantly elevated (p<0.05) (**Figure 11B**).

### 3.10 Identification and verification of BCHE as a potential biomarker

**Figure 12:**
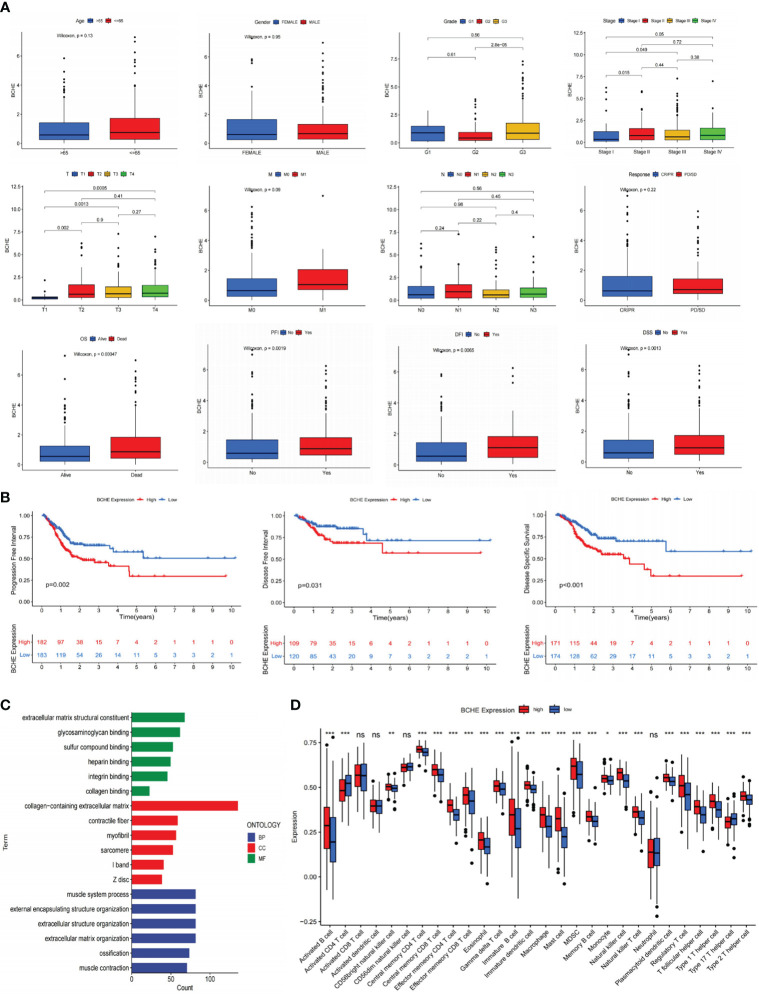
Clinicopathology, immune infiltration, and functional analyses of BCHE. **(A)** The correlation between BCHE and clinical pathological parameters of gastric cancer. **(B)** PFI, DFI, and DSS analyses in high- and low-BCHE expression subgroups. **(C)** Functional analysis of BCHE. **(D)** The relationship between BCHE and tumor-infiltrating immune cells. *P< 0.05; **P< 0.01; ***P< 0.001. PFI, Progression Free Interval; DFI, Disease Free Interval; DSS, Disease Specific Survival. ns: p>0.05.

To investigate the five genes in our signature in more detail, we plotted their 1, 3, and 5-year ROC curves and AUC values showed BCHE as the most prominent gene in the signature (**Figure 11C**). Consequently, the researchers made the decision to study BCHE in-depth. We first detected the protein levels of BCHE through immunohistochemistry (ICH) in the HPA database, and the findings indicated that the ICH staining level of BCHE in normal tissue was more pronounced than in gastric cancer tissue, and the staining site was mainly located in Cytoplasmic/membranous nuclear (**Figure 11D**). After that, we employed the TCGA database to perform a pan-cancer analysis on BCHE. Results demonstrated that BCHE was low expressed in the tumor tissues of BLCA, BRCA, LUAD, and LIHC, but considerably expressed in the tumor tissues of GBM, HNSC, and KIRP (**Figure 11E**). In order to explore the clinical relevance of BCHE even further, we examined its connection to the clinicopathological features of patients with gastric cancer. As evidenced by **Figures 12A, B**, BCHE is more pronounced in populations with lower differentiation (G3), later clinical and T-stages, death patients, as well as subgroups with shorter PFI, DFI, and DSS, indicating that BCHE may be an oncogene that can induce cancer development and lead to poor prognosis. Likewise, we conducted functional analysis on BCHE, and GO displayed that it is primarily related to muscle system process, extracellular structure, and matrix organization, as well as the binding of some compounds (**Figure 12C**). Additionally, BCHE is significantly related to the majority of TIICs, according to an assessment of its immune infiltration characteristics. BCHE expression exhibits a positive relationship with other TIICs but a negative correlation with activated CD4 T cells (**Figure 12D**).

**Figure 13:**
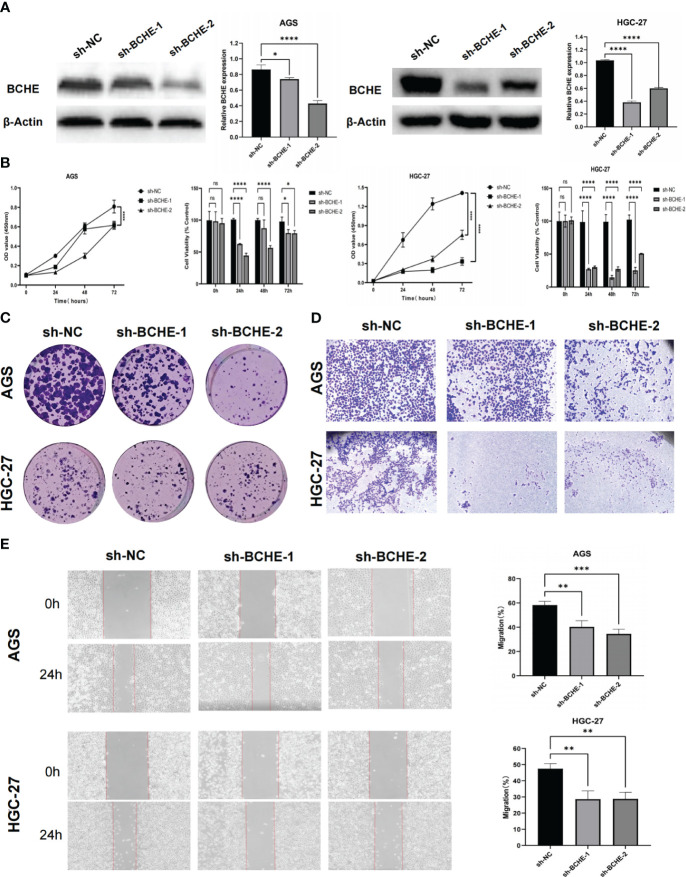
Verification of the tumor-promoting effect of BCHE in gastric cancer. **(A)** The expression level of BCHE control and knockdown groups in the AGS and HGC-27 cell lines was measured using Western blot. **(B)** The CCK-8 assay was used to access the proliferation and viability of BCHE in AGS and HGC-27 cell lines. **(C)** Using colony formation experiments to verify the effect of BCHE on the proliferation of AGS and HGC-27 cell lines. **(D)** Transwell assay was used to verify the role of BCHE in the migration of AGS and HGC-27 cell lines. **(E)** The wound healing experiments to explore the migration effect of BCHE in AGS and HGC-27 cell lines, and calculate its migration rate. *P< 0.05; **P< 0.01; ***P< 0.001. ****P< 0.0001. ns: p>0.05.

To illustrate the tumor-promoting effect of BCHE in gastric cancer, we successfully constructed knockdown BCHE lentiviral vectors (sh-BCHE-1, sh-BCHE-2) and RNAi negative control (sh-NC) vectors. We transfected them into AGS and HGC-27 cell lines. The Western blot results confirmed the transfection effect and the knockdown effect of sh-BCHE-2 in AGS and sh-BCHE-1 in HGC-27 was quite substantial (**Figure 13A**). We next performed the CCK-8 and colony formation assays, and the findings exhibited that the viability and proliferation ability of gastric cancer cells in the knockdown (sh-BCHE) groups were considerably decreased compared to the control group (sh-NC) (**Figures 13B, C**). Additionally, researchers also carried out Transwell and wound healing experiments. The outcomes unambiguously indicated that the migration ability of gastric cancer cells in the sh-BCHE groups was markedly diminished (**Figures 13D, E**). Therefore, we can preliminarily infer that BCHE has a promoting effect on the growth and migration of gastric cancer.

## 4 Discussion

Gastric cancer is a malignant digestive tract tumor with a poor prognosis and high fatality rate. It ranks third among malignant tumors in China (36). Early-stage GC patients’ outcomes have dramatically improved in the last several years thanks to advancements in surgical and gastroscopy techniques (37). However, the primary treatment option for advanced GC is still chemotherapy, having a dismal prognosis and less than 20% survival rate for five years (38, 39). Lipid metabolic reprogramming is one of the most prominent biological processes involved in the initiation and development of cancer (40). Tumor cells modify their lipid-related metabolic pathways and nutritional structures to accommodate metastasis. Research has proclaimed that tumor cells can upregulate the PI3K/Akt/mTOR pathway, which enhances glucose uptake and aerobic glycolysis while also increasing the production of metabolites required for the synthesis of FA and cholesterol (41–43). Additionally, the PI3K/Akt/mTOR pathway upregulates SREBP-1 to promote lipogenesis gene transcription and enhance lipid metabolism (44). Moreover, tumor cells can affect the activity of stromal or immune cells through lipid metabolism (LM), forming immune escape and immunosuppression, leading to treatment resistance and cancer recurrence. All these factors encourage the development of cancer. Given this, reducing the available lipids of tumor cells and intervening in their lipid metabolism may provide new therapeutic opportunities for gastric cancer.

For several years, constructing prognostic signatures according to particular biological characteristics has become a standard method in cancer research. Therefore, in the present research, we comprehensively and systematically created a signature according to lipid metabolism-related genes, further analyzed its clinical significance, evaluated the TME landscape, and forecasted immunotherapy’s efficacy in GC patients. In the beginning, we identified 148 DELRGs using RNA-seq data from the TCGA-STAD database and LRGs from the MsigDB website. GO and KEGG analyses revealed enrichment of these genes in lipid metabolism and carcinogenesis pathways. Subsequently, we screened 17 prognostic-related genes through univariate Cox analysis and identified their frequent CNV alterations, confirming that LM plays an essential role in GC lesions. Next, LASSO and multivariate Cox analyses were employed to create a suitable prognosis signature related to LM, and its excellent reliability and stability were verified in the GSE15459 cohort. According to K-M curves, patients in the LR subgroup exhibited noticeably longer OS and PFS (p<0.001), and the 5-year ROC curve (AUC value>0.70) demonstrated that our signature was satisfactory in forecasting prognosis. In the TCGA-STAD queue, relations between the signature and specific clinicopathological parameters (like age, gender, survival status, grade, T, N, and M stages) were analyzed. The outcomes indicated that the high-risk category had more severe clinical characteristics and worse prognoses for GC patients. Both univariate and multivariate Cox analyses confirmed that LRGs-based risk scores were a reliable predictor of GC prognosis. We then developed a nomogram to improve our signature’s clinical applicability. This nomogram has a certain predictive effect on the prognosis of GC individuals at 1-, 3-, and 5- years and exhibited optimal predictive capacities. In addition, GSEA analysis applied that the LR group mainly was linked to metabolic processes, while the HR group enriched stromal-activated and tumor-associated signaling pathways more, including Focal adhesion, cell adhesion molecules CAMs, PPAR, Wnt, Calcium, and TGF beta signaling. This could be connected to the dismal prognosis for the group with high-risk scores.

Afterward, we performed landscape analysis on the signature tumor microenvironment (TME) and observed that the HR group had elevated stromal activity. Previous studies have indicated that stromal cells can accelerate the growth and dissemination of tumors by inhibiting immune cells from penetrating and entering the tumor parenchyma, preventing T cells from killing tumor cells, and inducing angiogenesis (45–48). Furthermore, our research demonstrated positive correlations between risk scores and the infiltration of most TIICs, especially regulatory T cells (Tregs), myeloid suppressor cells (MDSC), and macrophages, suggesting that high levels of infiltration of these cells exist in populations with high-risk scores. Tregs are a highly immunosuppressive subset of CD4+ T cells and as a gatekeeper for immunological homeostasis, suppressing effective anti-tumor immunity through different mechanisms (49). Notably, tumor-associated macrophages (TAMs) are typically the most abundant myeloid cells in different TMEs. TAMs have high functional plasticity and can affect many tumor processes, including immunosuppressive TME formation, tumor angiogenesis activation, and ECM remodeling (49–51). It is reported that cholesterol efflux from cells can encourage TAMs to polarize towards the M2-like phenotype, often associated with worse prognosis and poor treatment response in human malignancies (52). Besides, 27-HC (one of the primary metabolites of cholesterol) can promote the differentiation of MDSCs, inducing immune escape of tumor cells (53).

With the arrival of the era of immunotherapy, the outcome of gastric cancer individuals has significantly improved. Pembrolizumab (PD-1 inhibitor) is the world’s first immunotherapy approved for treating advanced solid tumors in the MSI-H/dMMR state. According to KEYNOTE-158 global data (K cohort), the objective response rate (ORR) of gastric or gastroesophageal junction cancer patients is 39% (54). In Chinese patients (L cohort), the ORR is as high as 63%, indicating that the MSI-H/dMMR population in China can achieve more prominent benefits from the treatment of pembrolizumab. However, the effectiveness of immunotherapy differs in various groups due to individual characteristics, and determining which populations are more probable to benefit from immunotherapy is therefore crucial. Somatic mutations have been proven to participate in immunotherapy by generating tumor-specific neoepitopes (55). After removing germline mutations, the total somatic mutations in the tumor genome are known as tumor mutation burden (TMB), which can activate CD8+cytotoxic T cells and initiate T cell-mediated anti-tumor effects (56). Theoretically, the greater the TMB values, the more neoepitopes can be identified by T cells and the superior immunotherapy efficacy. As a result, we examined somatic mutations and TMB in two risk subgroups, detecting that the LR group exhibited a significantly higher mutation rate and TMB scores. Another important discovery was that the low-risk category’s TIDE, Dysfunction, and Exclusion scores were considerably lower, while the expression of PD-L1 and MSI-H was much more remarkable. This suggested that individuals in the low-risk subgroup may experience less immune escape and be more sensitive to immunotherapy. More importantly, in the joint analysis of ICIs (anti-PD-1 and anti-CTLA-4 antibodies), immunotherapy will provide greater therapeutic benefit to low-risk GC patients, leading to superior outcomes. According to drug sensitivity analysis, the low-risk group also showed a lower IC50 for several commonly used chemotherapeutic and targeted drugs. This indicated that persons in the low-risk category were better responsive to these medications and had a greater likelihood of benefiting.

In this study, researchers recognized five genes that serve as risk signature genes- APOA1, BCHE, CYP19A1, PLA1A, and STARD5. Apolipoprotein A1 (APOA1) is a crucial component of high-density lipoprotein (HDL) and an essential cofactor of cholesterol transferase (LCAT) (57). LCAT can catalyze the production of cholesterol esters and lysophosphatidic from free cholesterol in the plasma. In addition, the increase of HDL/ApoA1 in plasma can prevent the progression of diabetes (58), nervous system disease (59), and inflammation (60, 61), as well as play a protective role in atherosclerosis and related cardiovascular diseases (62, 63). Significantly, APOA1 is specifically and negatively correlated with survival in various solid tumor forms, including colorectal, breast, and esophageal (64–66). Furthermore, research has demonstrated that APOA1 expression in small cell lung cancer (SCLC) is higher than in normal lung tissue or non-small cell lung cancer (NSCLC), and that APOA1 expression is significantly lower in patients with recurrent SCLC and those who underwent neoadjuvant chemotherapy prior to surgery (67). Consequently, APOA1 may have significant potential as an anti-tumor drug. Butyrylcholinesterase (BCHE) is a non-specific esterase synthesized by the liver. It contributes to the inactivation of the neurotransmitter acetylcholine and degrades neurotoxic organophosphate esters. Previous studies on BCHE have mostly focused on Alzheimer’s disease (AD) and inhibiting the expression of BCHE can potentially treat new symptoms of AD (68, 69). Additionally, BCHE is associated with tumorigenesis, cell proliferation, and differentiation (70). It is highly expressed in breast, oral, and ovarian cancers (70–72) but has low expression in colorectal and endometrial cancer (73, 74). In prostate cancer, BCHE expression is down-regulated early on and up-regulated in the late stage (75). Cytochrome P450 family 19 subfamily A member 1 (CYP19A1) can catalyze the conversion of androgen/testosterone to estradiol/estrone, the main enzyme involved in estrogen production (76). Breast cancer, endometrial cancer, and Alzheimer’s disease are intimately associated with CYP19A1 (77–79). The research has pointed out that it is prominently increased in human colon cancer tissue and mediated immunosuppression through the GPR30-Akt pathway, promoting colon cancer progression and chemotherapy resistance (80). Phospholipase A1 member A (PLA1A) is a type of pancreatic lipase expressed in multiple tissues and organs of the human body, which regulates the maturation and function of numerous immune cells (81). PLA1A can promote tumor progression via activating the PI3K/Akt pathway induced by GPR34 (82) or converting lysozyme PS into LPA (a lipid mediator related to cancer progression and metastasis) mediated by ATX (83, 84). The invasion, metastasis, and worse prognosis of colorectal, glioma, and prostate cancer correlate with elevated PLA1A expression (81, 85, 86). Furthermore, PLA1A is a potential diagnostic marker for advanced and BRAF mutant melanoma (87). The STARD4 subfamily, which STARD5 is a member of, consists primarily of a START domain without a specific organelle targeting sequence. Several physiological processes, including lipid transport and metabolism, signal transduction, and transcriptional control, are associated with the START domain (88). Research has shown that STARD5 expression is induced upon endoplasmic reticulum (ER) stress and participates in regulating cholesterol balance. Abnormal cholesterol metabolism is directly correlated with tumor occurrence and growth. It has been established that STARD5 is a valuable biomarker for assessing hepatocellular carcinoma (HCC) prognosis, and high expression of STARD5 implies a better prognosis (89).

Based on the comprehensive ROC curves and qRT-PCR expression, we have decided further to explore the role of BCHE in gastric cancer progression. Patients with high BCHE expression have later stages, lower differentiation (G3), shorter OS, PFS, and DFS. Through CCK-8, colony formation, Transwell, and wound healing assays, we preliminarily infer that BCHE can promote the growth and migration of gastric cancer cells.

It’s critical to recognize the limitations of this study. Even though we used bioinformatics analysis to create a prediction signature based on lipid metabolism and verified it using the GEO database and partial cell experiments, more experimental confirmation is still needed. Then, although we have preliminarily demonstrated the promoting effect of BCHE on the growth and migration of gastric cancer, further exploration of its mechanism of action is still needed. Furthermore, while this signature has demonstrated considerable promise in predicting the response to immunotherapy, it needs validation in a cohort of GC patients receiving immunotherapy. Therefore, an in-depth study is essential to comprehend the precise mechanism underlying this predictive model.

## 5 Conclusions

In conclusion, our study successfully constructed a risk signature based on lipid metabolism-related genes that can independently predict prognosis and develop a nomogram to improve its clinical practicality. In addition, this study elucidated the relationship between lipid metabolism and immune infiltration characteristics and predicted the immunotherapy efficacy of GC patients based on risk scores. By using bioinformatics analysis and partial cell experiments, we were able to establish that BCHE is an oncogenic gene and to identify it as a potential biomarker for gastric cancer. Briefly, this investigation offers new directions for locating viable prognostic biomarkers and more efficient treatment options for GC.

## Data availability statement

The datasets presented in this study can be found in online repositories. The names of the repository/repositories and accession number(s) can be found in the article/**Supplementary Material**.

## Ethics statement

Ethical approval was not required for the studies on humans in accordance with the local legislation and institutional requirements because only commercially available established cell lines were used.

## Author contributions

SiW: Data curation, Formal analysis, Methodology, Writing – original draft, Writing – review & editing, Software. XH: Validation, Methodology, Writing – review & editing, Writing – original draft. RW: Formal analysis, Methodology, Software, Writing – review & editing, Data curation, Writing – original draft. SZ: Data curation, Funding acquisition, Writing – review & editing. JL: Data curation, Formal analysis, Writing – review & editing. YL: Resources, Visualization, Writing – review & editing. SaW: Data curation, Formal analysis, Writing – review & editing. JG: Formal analysis, Methodology, Writing – review & editing. YW: Validation, Writing – original draft. MZ: Data curation, Formal analysis, Writing – review & editing. WQ: Formal analysis, Investigation, Methodology, Writing – review & editing.
